# Repeating late-phase pseudo-progression in a patient with non-small cell lung cancer treated with long-term nivolumab monotherapy; a case report

**DOI:** 10.3389/fonc.2024.1353698

**Published:** 2024-07-08

**Authors:** Rikako Ebisuda, Naoki Furuya, Takeo Inoue, Shotaro Kaneko, Yu Numata, Yusuke Shinozaki, Masamichi Mineshita

**Affiliations:** Division of Respiratory Medicine, Department of Internal Medicine, St. Marianna University School of Medicine, Kawasaki, Japan

**Keywords:** pseudo-progression, nivolumab, non-small cell lung cancer, lymph node enlargement, long-term response, long-term survival

## Abstract

**Background:**

Immune check point inhibitors (ICIs) are standard treatment for patients with non-small cell lung cancer (NSCLC). Nearly a decade has passed since nivolumab was approved by the FDA for NSCLC patients. However, long-term outcomes and clinical features remain unclear for individual cases. Pseudo-progression is a well-known paradoxical radiological response pattern under ICI treatment which occurs when tumor index lesions regress after apparent initial progression. We herein report a unique case of NSCLC with repeating pseudo-progression in late phase treated with nivolumab monotherapy for 8.5 years.

**Case presentation:**

A 56-year-old male diagnosed with Non-sq NSCLC clinical stage IVA, at the left upper lobe primary lesion. The primary lesion was PD-L1 negative with no oncogenic driver mutations. He had multiple pulmonary metastases and a left adrenal gland metastasis, and subsequently, received nivolumab as third-line therapy. After initiation of nivolumab, the lung lesion and adrenal metastasis shrank rapidly; however, the patient experienced three late-phase pseudo-progressions in the mediastinal lymph node (LN). This patient is still receiving nivolumab with no symptoms and PS 0. Acquired resistance should be judged carefully in patients with LN-only oligo-progression to avoid unnecessary local therapies and the misjudgment of treatment.

## 1 Introduction

Immune check point inhibitors (ICIs) are standard treatment for patients with non-squamous non-small cell lung cancer (Non-sq NSCLC) (1). Currently, ICIs are not only used for advanced stages, but also for early-stage resectable NSCLC (2). Nivolumab was the first anti-programmed death-1 (PD-1) antibody which was approved for NSCLC by the FDA; however, long-term outcomes and clinical features are unclear for individual cases - especially in long-time durable responders and survivors.

Pseudo-progression is a well-known paradoxical radiological response pattern under ICI treatment which is not observed in conventional cytotoxic chemotherapy (3). Pseudo-progression occurs when tumor index lesions regress after initial progression. The frequency of pseudo-progression was reported 3.6 - 6.9% in NSCLC patients treated with ICI monotherapy (4). We herein report a unique case of repeating late-phase pseudo-progression in a NSCLC patient treated with nivolumab monotherapy for 8.5 years.

## 2 Case report

The patient was a 56-year-old male diagnosed with clinical stage cT3N0M1b IVA (the 8th edition of UICC) non-squamous NSCLC. The primary lesion was located at the left upper lobe with multiple pulmonary metastases and a left adrenal gland metastasis. He was a heavy smoker (45 pack-years) with well-controlled type 2 diabetes and no family history of cancer. We pathologically diagnosed lung adenocarcinoma by CT-guided core needle biopsy sample which was obtained from the primary lesion. PD-L1 was negative (22C3 PharmDx, Dako), and no oncogenic driver mutations, such as *EGFR*, *ALK*, *ROS1*, *RET* and *BRAF* were detected at the time of initial diagnosis by PCR-based testing, IHC and FISH. At initial diagnosis, this patient was suffering from intense, constant and radiating pain at the chest wall, in addition to left arm and hand numbness. These symptoms were not well-controlled, even after treatment with NSAIDs and high-dose opioid. Therefore, the presenting radiation oncologist considered intense localized treatment for the superior sulcus tumor (Pancoast tumor).

The first-line treatment was concurrent chemo-radiotherapy (CCRT) with cisplatin plus docetaxel and 64 Gy radiotherapy to left upper lobe Pancoast tumor. The best response was partial response (PR). Twenty-four months after CCRT, the left adrenal gland metastasis was enlarged and the patient received a combination of carboplatin and pemetrexed, followed by pemetrexed maintenance therapy as second-line treatment. The best response was PR.

**Figure 1:**
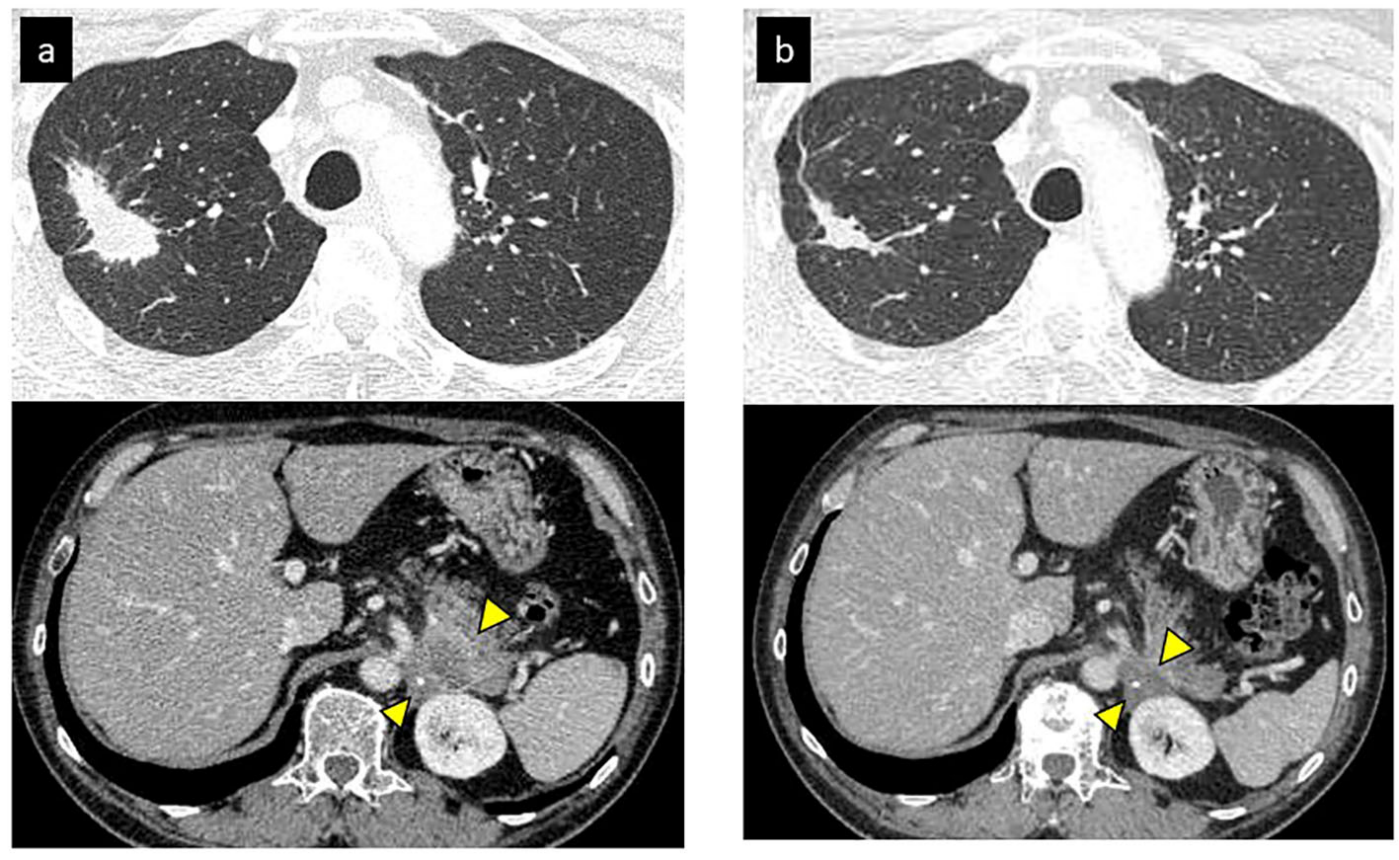
Initial tumor response of nivolumab (partial response). **(A)** CT revealed a primary lesion in right upper lobe and metastases of left adrenal gland before nivolumab. **(B)** Primary lesion and adrenal gland metastasis shrunk after 2 months of nivolumab.

Thirty-nine months after initiation of second line-treatment, the patient complained of lower back pain. CT image showed a pulmonary metastasis lesion in the right upper lobe and the left adrenal gland were enlarged (**Figure 1A**). He presented with Eastern Cooperative Oncology Group (ECOG) performance status (PS) 1, and his tumor markers were elevated (CEA: 124.6 ng/mL). At that time, December 2015, nivolumab was approved for patients with NSCLC in Japan. We initiated nivolumab as third-line therapy at a dose of 3 mg/kg bodyweight every two weeks.

**Figure 2:**
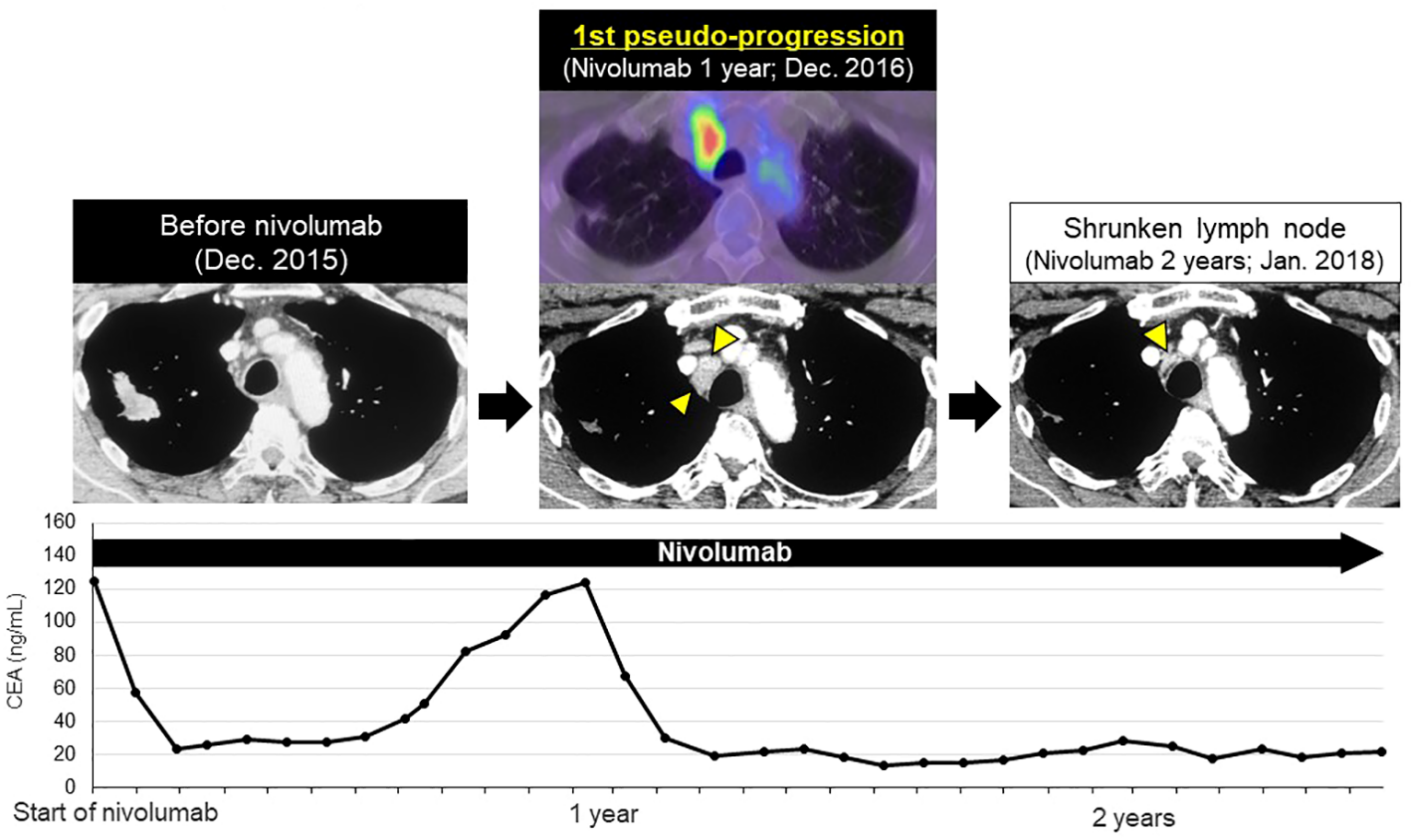
First late-phase pseudo-progression after 1-year of nivolumab. After 7-months of nivolumab, tumor markers increased. CT scan at 1-year showed swollen mediastinal lymph node (LN, short axis 15 mm, 1st pseudo-progression; new measurable LN lesion), PET-CT scan revealed a high-level uptake of the mediastinal LN. At 2-years, the mediastinal LN shrunk significantly (short axis 4 mm).

After initiation of nivolumab, the right upper lobe lesion and adrenal metastasis shrunk rapidly (**Figure 1B**), and his tumor markers decreased (**Figure 2**). ECOG PS improved from PS 1 to PS 0. Based on the Response Evaluation Criteria in Solid Tumors (RECIST), the best response was PR. Although grade 1 pruritus occurred during nivolumab, no treatment was necessary. However, 7 months later, the patient’s tumor markers gradually increased (**Figure 2**). A subsequent CT scan 1 year after initiation of nivolumab revealed a swollen mediastinal lymph node (LN), (short axis 15mm). Positron emission tomography-computed tomography (PET-CT) scan showed a high-level uptake for the mediastinal LN. We diagnosed the patient with acquired resistance (LN oligo-progression) and progressive disease (PD) to nivolumab based on RECIST criteria. However, ECOG PS remained at PS 0, and the patient hoped to continue nivolumab. We considered stereotactic body radiotherapy (SBRT) for the mediastinal LN oligo-progression site; however, we had already irradiated the same thoracic field during first-line treatment. We continued nivolumab since there were no other established standard treatments for this patient, except for low effective cytotoxic chemo-monotherapies.

After RECIST PD diagnosis, his tumor markers gradually decreased, and the mediastinal LN shrank (**Figure 2**). We clinically diagnosed the elevated tumor markers and LN swelling as atypical late-phase pseudo-progression under nivolumab. However, 3.5-years after the initiation of nivolumab, tumor markers increased and the mediastinal LN was again swollen (short axis 5 to 27 mm, 2nd pseudo-progression; +440%). PET-CT scan revealed a high-level uptake for the mediastinal LN.

**Figure 3:**
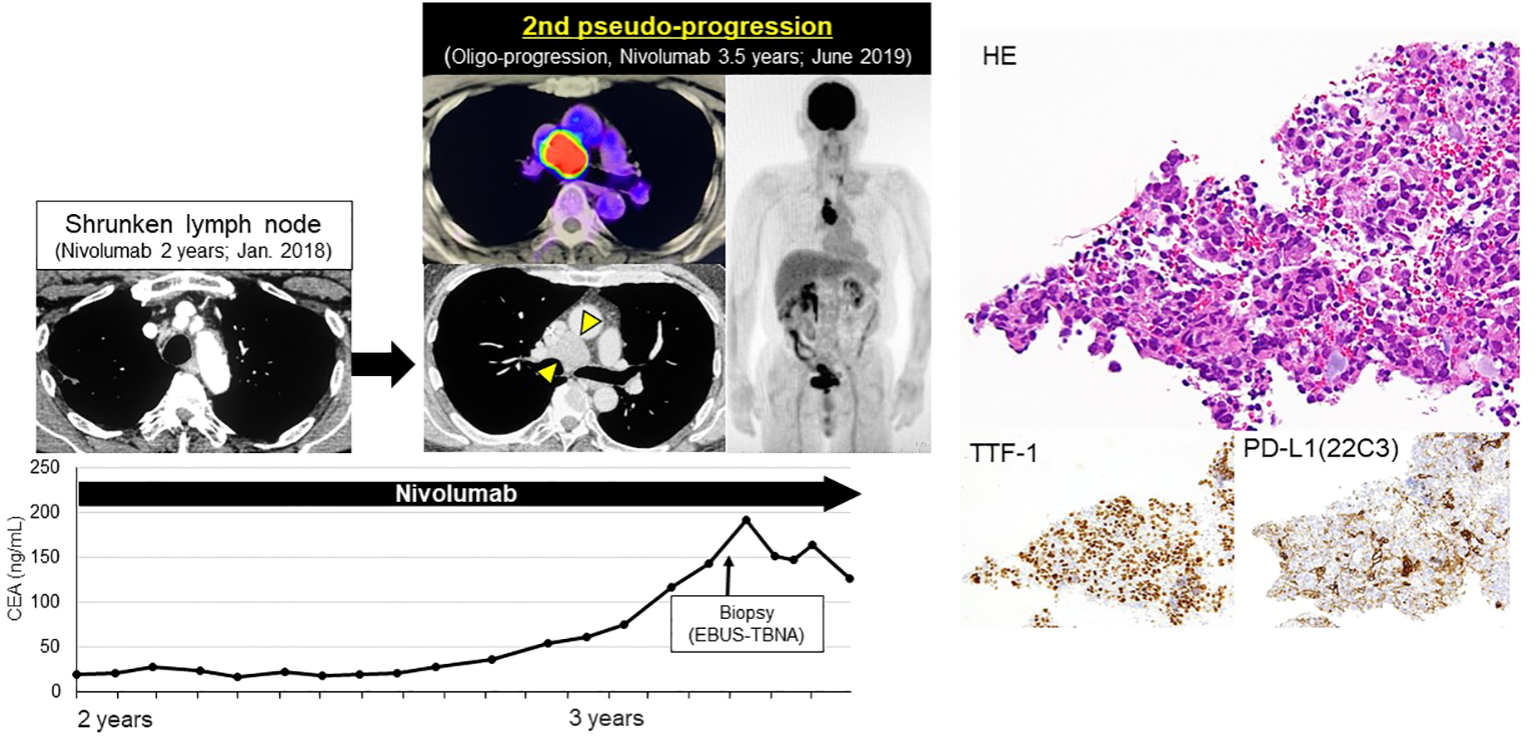
Second late-phase pseudo-progression at 3.5-years of nivolumab. At 3.5-years of nivolumab, tumor markers again increased and mediastinal LN was swollen (short axis 5→27 mm, 2nd pseudo-progression; +440%). PET-CT scan revealed a high-level uptake of the mediastinal LN. LN specimens obtained by EBUS-TBNA revealed TTF-1 positive tumor cells and lymphocytes. PD-L1 TPS was 10%.

**Figure 4:**
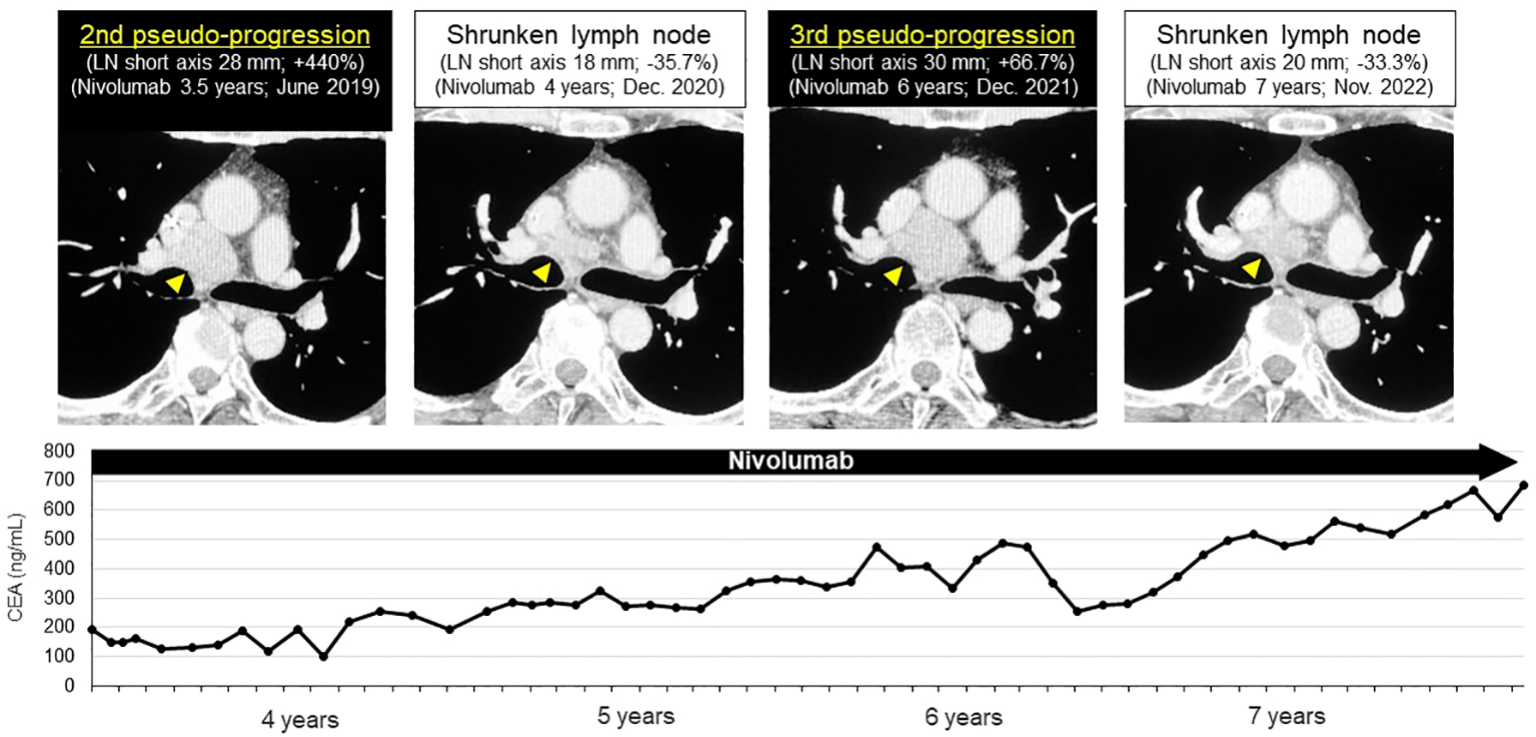
Third late-phase pseudo-progression at 6-years of nivolumab. After biopsy, the lymph node shrunk again. Third pseudo-progression was observed after 6-years of nivolumab. Pseudo-progression repeated three-times in this patient during nivolumab administration (short axis 18→30 mm, 3rd pseudo-progression; +66.7%).

We confirmed the pathological diagnosis of the mediastinal LN #4R by endobronchial ultrasound sonography-transbronchial needle aspiration biopsy (EBUS-TBNA). From #4R LN specimen, metastatic LN was diagnosed pathologically with TTF-1 positive tumor cells and some lymphocytes. PD-L1 stain showed the tumor proportion score (TPS) as 10% immunohistochemically (22C3, **Figure 3**). Next generation sequencing-based multi-gene panel (Oncomine Precision Assay, 50 gene mutation and fusion; Thermo Fisher Scientific) was performed for molecular testing. No oncogenic driver alterations, including *EGFR*, *KRAS*, *HER2*, *BRAF*, *MET ex14 skipping* and fusion genes (*ALK*, *ROS1*, *RET and NTRK*) were detected. Even though pathological confirmation was obtained, we continued to administer nivolumab since the primary lesion and adrenal metastasis were well-controlled, except for the mediastinal #4R LN. After biopsy, the LN shrunk again, therefore, we diagnosed the patient with a second pseudo-progression. Interestingly, a third pseudo-progression was observed after 6 years of nivolumab (short axis 18 to 30 mm, 3rd pseudo-progression; +66.7%). Although the patient experienced a total of three pseudo-progressions (**Figure 4**), he has been receiving nivolumab monotherapy for 8.5 years with no symptoms and PS 0.

## 3 Discussion

Here, to the best of our knowledge, we describe the first case of a NSCLC patient with repeating pseudo-progression in late phase who was treated with long-term nivolumab monotherapy. When nivolumab was approved for advanced stage melanoma and NSCLC, pseudo-progression was reported as the atypical radiological response and clinical feature in early phase, within one or two months after initiation of nivolumab. Interestingly, in this patient, pseudo-progressions were observed a total of three times in the late phase. If physicians are unaware of this phenomenon, they might misjudge acquired resistance and genuine progressive disease (PD). This could result in switching treatment regimen or additional local therapy.

The CheckMate-017/057 study, a long-term follow-up report, revealed that 38% of patients (19/50) were judged PD to nivolumab based on the RECIST criteria, which included 5-year survivors (5). These patients received other cytotoxic chemotherapies, retreatment of nivolumab or local therapies. Gettinger et al. reported clinical features of 26 NSCLC patients with acquired resistance to anti-PD-1 inhibitor monotherapy (6). The report revealed 77% (20/26) of acquired recurrence sites were LNs, including 42.3% (11/26) LN-only progression, referred to as “oligo-progression”. In Gettinger’s report, some patients with acquired resistance who were judged by RECIST criteria v1.1 or immune-related response criteria, received local therapy including surgery and radiotherapy in clinical practice.

Some previous reports suggest that host immune status and external factors such as COVID-19 vaccinations (7), corticosteroids (8), and antibiotics/probiotics (9) affect anti-cancer immune systems by stimulating or suppressing immune cells. It is well known that diet and lifestyle also affect immune status, these potentially influence the efficacy for ICI therapy (10). In this patient, we did not change the dose of nivolumab, nor did this patient receive any vaccinations or additional drugs during the three pseudo-progressions. Furthermore, smoking cessation had already been achieved in this patient before initiation of nivolumab.

The mechanism of pseudo-progression remains unclear, but one of the mechanisms may be associated with tissue edema and tumor necrosis due to inflammatory cell infiltration and cytokines (11). In this patient, significant infiltration of lymphocytes and macrophages were seen around tumor cells in the second biopsy specimens (**Figure 3**). Interestingly, PD-L1 upregulated from 0% to 10% after nivolumab treatment. We considered following two reasons, i) intra-tumoral and intra-patient heterogeneity (12), ii) dynamic change due to prior treatment (13). In this patient, initial biopsy site was left upper lobe primary lesion, second biopsy site was mediastinal LN #4R. And this patient received prior chemo-radiotherapy before nivolumab treatment. These factors might influence the discordance between initial biopsy sample and second biopsy.

This case report included several limitations. Firstly, we were unable to identify the exact date of the pseudo-progressions since chest CT and PET scans were not performed frequently or at the same intervals during nivolumab treatment. Secondly, we did not perform immunological evaluation for re-biopsy sample to investigate the mechanisms of pseudo-progression by IHC or single cell RNA sequencing. Thirdly, since repeating late-phase pseudo-progression is rare, there is limited literature on this phenomenon. Therefore, larger retrospective studies and case series are needed.

In summary, repeating late phase pseudo-progressions were observed in this patient treated with long-term anti-PD-1 therapy. Acquired resistance should be judged carefully in patients with LN-only oligo-progression to avoid unnecessary local therapies and the misjudgment of treatment.

## Data availability statement

The original contributions presented in the study are included in the article/supplementary material. Further inquiries can be directed to the corresponding author.

## Ethics statement

Written informed consent was obtained from this patient for the publication of any potentially identifiable images or data included in this article. The studies were conducted in accordance with the local legislation and institutional requirements. The participants provided their written informed consent to participate in this study. Written informed consent was obtained from the participant/patient(s) for the publication of this case report.

## Author contributions

RE: Conceptualization, Data curation, Writing – original draft, Writing – review & editing. NF: Conceptualization, Data curation, Investigation, Supervision, Writing – original draft, Writing – review & editing. TI: Conceptualization, Investigation, Writing – review & editing. SK: Conceptualization, Writing – review & editing. YN: Conceptualization, Writing – review & editing. YS: Conceptualization, Writing – review & editing. MM: Conceptualization, Investigation, Supervision, Writing – review & editing.
